# 

**Published:** 1892-12

**Authors:** 


### Southern Medical Record

A Monthly Journal of Medicine and Surgery.

VOL. XXII. ATLANTA, GA., DECEMBER, 1892. NO. 12.

EDITORS:

A. W. GRIGGS, M. D. WILLIS F. WESTMORELAND, M. D.

J. McFADDEN GASTON, M. D. DAN. H. HOWELL, M. D., Bus. Mgr.

TERMS: $2.00 Per Annum; Single Copy, 20 Cents.

Contents on Page 5.

Entered at the Postoffice at Atlanta, Ga., as Second-Class Matter.

Postell & Sexton, Publishers. 23 S. Broad, Atlanta. Ga.

